# Stable, Room-Temperature, Low-Threshold Amplified Spontaneous Emission from Thermally Evaporated Cesium Lead Halide Perovskites

**DOI:** 10.1021/acsnano.5c03771

**Published:** 2025-07-16

**Authors:** Yuliia Kominko, Sebastian Sabisch, Andrii Kanak, Lidiia Dubenska, Ihor Cherniukh, Matthias Klimpel, Xuqi Liu, Sergey Tsarev, Simon C. Boehme, Gebhard J. Matt, Gabriele Rainò, Maksym V. Kovalenko, Sergii Yakunin

**Affiliations:** † 27219 ETH Zürich, Department of Chemistry and Applied Biosciences, Laboratory of Inorganic Chemistry, Vladimir-Prelog-Weg 1, Zürich CH-8093, Switzerland; ‡ Empa–Swiss Federal Laboratories for Materials Science and Technology, Laboratory for Thin Films and Photovoltaics, Überlandstrasse 129, Dübendorf CH-8600, Switzerland

**Keywords:** amplified spontaneous emission, lead halide perovskites, thermal evaporation, nanomaterials, thin films

## Abstract

Semiconductor nanocrystals are widely investigated as tunable quantum emitters due to their composition-, size- and shape-dependent optical properties, and they find applications in various optoelectronic devices, including lasers. However, in compact films, insulating ligands often limit heat and charge transport. Improving the heat management, for example, by removing ligands, can significantly enhance the operational stability at high excitation densities required for laser devices. Here, we report the synthesis, structural characterization, and stable high optical gain of ligand-free nanocrystalline thin films obtained through single-source thermal evaporation of CsPbX_3_ (X = Cl, Br). This deposition method is robust, scalable, and compatible with industrial processes. A slow postdeposition crystallization process under nitrogen yields compact, smooth, and optically uniform thin films with nanocrystalline grains with weakly confined optical features and pronounced excitonic resonances. The films show composition-tunable (430–545 nm), low-threshold (*ca*. 2 μJ cm^–2^) amplified spontaneous emission, with a high net modal gain of 890 cm^–1^ measured for the pure CsPbBr_3_ composition. Due to the enhanced heat dissipation enabled by the optimized film morphology, the operation stability under ambient conditions surpasses 180 million laser shots (*i*.*e*., 5 h of continuous operation), with merely ∼8% degradation, indicating that thermally evaporated perovskite thin films are promising optical gain media for room-temperature lasing applications.

## Graphical abstract

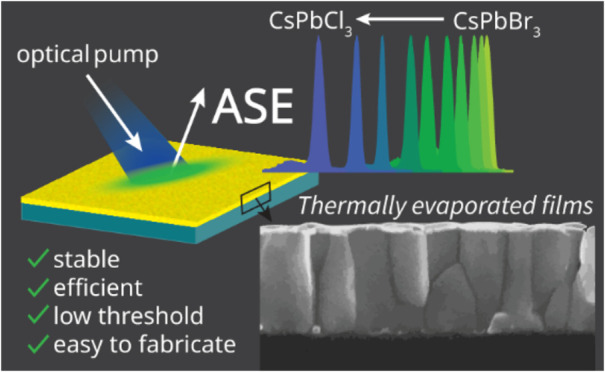

Lead halide perovskites (LHPs) are an emerging class of semiconductor materials that have garnered significant interest over the past decade due to their exceptional optoelectronic properties. These include scalable and cost-efficient synthesis at low temperatures and pressures, often even under ambient conditions, wide spectral tunability over the visible spectrum, spectrally narrow emission, high absorption coefficients, large charge-carrier diffusion lengths, long carrier lifetimes, and defect tolerance.^1–7^ Combining these characteristics promotes LHPs as a novel material platform for solar cells, hard radiation detectors, light-emitting diodes, and lasers.^8–13^ The general composition of LHPs is APbX_3_, where A is a cation such as Cs^+^, methylammonium (MA^+^), formamidinium (FA^+^), or aziridinium (AZ^+^), and X is a halide (Cl^–^, Br^–^, or I^–^), resulting in a three-dimensional (3D) network of corner-sharing PbX_6_ octahedra. Compared to commonly employed inorganic semiconductors such as Si or GaAs, the low formation energy renders LHPs structurally soft semiconductors.^14,15^

While light-harvesting devices such as photovoltaic solar cells have primarily considered bulk forms of LHPs, *e*.*g*., polycrystalline thin films or single crystals, light-emitting devices have increasingly employed nanocrystalline forms of LHPs.^16,17^ Especially when prepared in the form of colloidal nanocrystals (CNCs), LHP nanostructures exhibit near-unity photoluminescence quantum yield (PLQY), high color purity, tunable bandgaps across the visible spectrum *via* size or halide composition from blue-emitting CsPbCl_3_ to green CsPbBr_3_ and red CsPbI_3_ or any color in between, as well as solution processability.^18–22^ The LHP CNCs are of particular interest for wavelength-tunable amplified spontaneous emission (ASE) and lasing applications.^13^ While significant progress has been made over the past decade, further optimization of long-term operational stability under high excitation densities remains a challenge. For example, the ASE intensity of surface-treated CsPbBr_3_ CNC films decreases to 75% of the initial value after 5 million laser shots (*i*.*e*., 1.3 h of operation).^23^ Operational instability is commonly attributed to poor local heat management associated with the presence of long and insulating organic ligands.^24–26^ These molecules primarily consist of aliphatic chains and are essential for synthesizing high-quality colloids. Consequently, strategies for improved heat management, especially under high excitation densities, need to be established.

While the use of shorter ligands, for example, through solid state ligand exchange, could yield improvements, solution-processed LHP thin films can be assumed as a viable alternative. They circumvent the need for ligands while preserving high absorption coefficients, nano- and microcrystalline morphologies, and a high defect tolerance,^27^ and even enabled the first observation of continuous-wave lasing in LHPs.^28^ In practice, however, CsPbX_3_ thin films are particularly prone to macroscopic defects, including voids, pinholes, and extended surfaces with dangling bonds not passivated by ligands. Nonradiative recombination is thus promoted, significantly suppressing the ASE performance^29^ and reducing stability. For example, reported solution-processed phase-stable mixed-cationic LHPs experience a 5% drop in ASE intensity after 10 min of operation at 80 K.^28^

This study presents an efficient optical gain medium obtained through single-source thermal evaporation^17,30–35^ of melt-grown and ground CsPbX_3_ crystals.^36–38^ Compared to previous works, the presented method leverages the low formation energy to control the growth of amorphous material into defined nanocrystalline grains without requiring any additional influence on the sample, such as solvent treatment, pressurization, sample heating, or substrate-specific treatments.^39–46^ The resulting compact films comprising ligand-free nanocrystalline grains, with preferential crystallographic orientation, exhibit stable ASE under ambient conditions at high excitation densities. The peculiar morphology stems from the slow room-temperature (RT) recrystallization, under nitrogen, of the initially formed amorphous films. The film evolution was monitored using atomic force microscopy (AFM), as well as absorption and photoconductivity spectra. While the grain sizes of the evaporated films, observed by AFM and scanning electron microscopy (SEM), appear too large to induce excitonic quantum confinement, nuclear magnetic resonance (NMR) studies reveal a structural disorder comparable to that of CNCs. The crystallized films demonstrate outstanding performance as an optical gain medium, outperforming previously reported works by combining the ASE threshold, the net modal gain, and operational stability.^13,28,39,47–50^ The evaporated LHP thin films reveal spectrally tunable (430–545 nm) and low thresholds (2–6 μJ cm^–2^) ASE, with a net modal gain of up to 890 cm^–1^ for the pure CsPbBr_3_ composition. The ASE remains stable under ambient conditions and high excitation density for over 180 million laser shots (*i.e*., enduring 5 h of continuous operation) exhibiting only *ca*. 8% loss in intensity, thereby surpassing the operational stability reported in previous reports^23,51^ or showing a lower ASE threshold while being on par with reported stability.^52,53^ The combination of the exceptional ASE threshold and enhanced operational stability represents a key advancement toward realizing thermally evaporated LHP thin films as viable platforms for optical amplification and lasing.^23,28,54,55^

## Results and Discussion

### Deposition and Crystallization of Thermally Evaporated Films

While conventional solution processing is widely employed for bulk LHP thin-film fabrication, thermal perovskite deposition offers notable advantages such as scalability, compatibility with substrates of diverse materials and shapes, and ease of automation, making it well-suited for integration into manufacturing processes (Figure S1). Once the starting material, namely, a powder made from high-purity melt-grown CsPbX_3_ crystal, is thermally deposited on a substrate, it forms an amorphous film (Figure 1a, left). With time, the amorphous film crystallizes into grains of the perovskite phase. The speed of this process and the resulting grain size depend on the temperature.^56–58^ The optimal film quality was achieved through slow crystallization at RT, which took a few days to a month, resulting in a compact film with a relatively narrow grain size distribution (Figure 1a, middle). Notably, the crystallization process was correlated with improved optical gain properties and decreased ASE thresholds, achieving optimal values after a month of sample aging at RT. Meanwhile, fast crystallization at elevated temperatures leads to the formation of perforated nonuniform films where some grains are consumed by the neighboring larger grains (Figure 1a, right).

**Figure 1:**
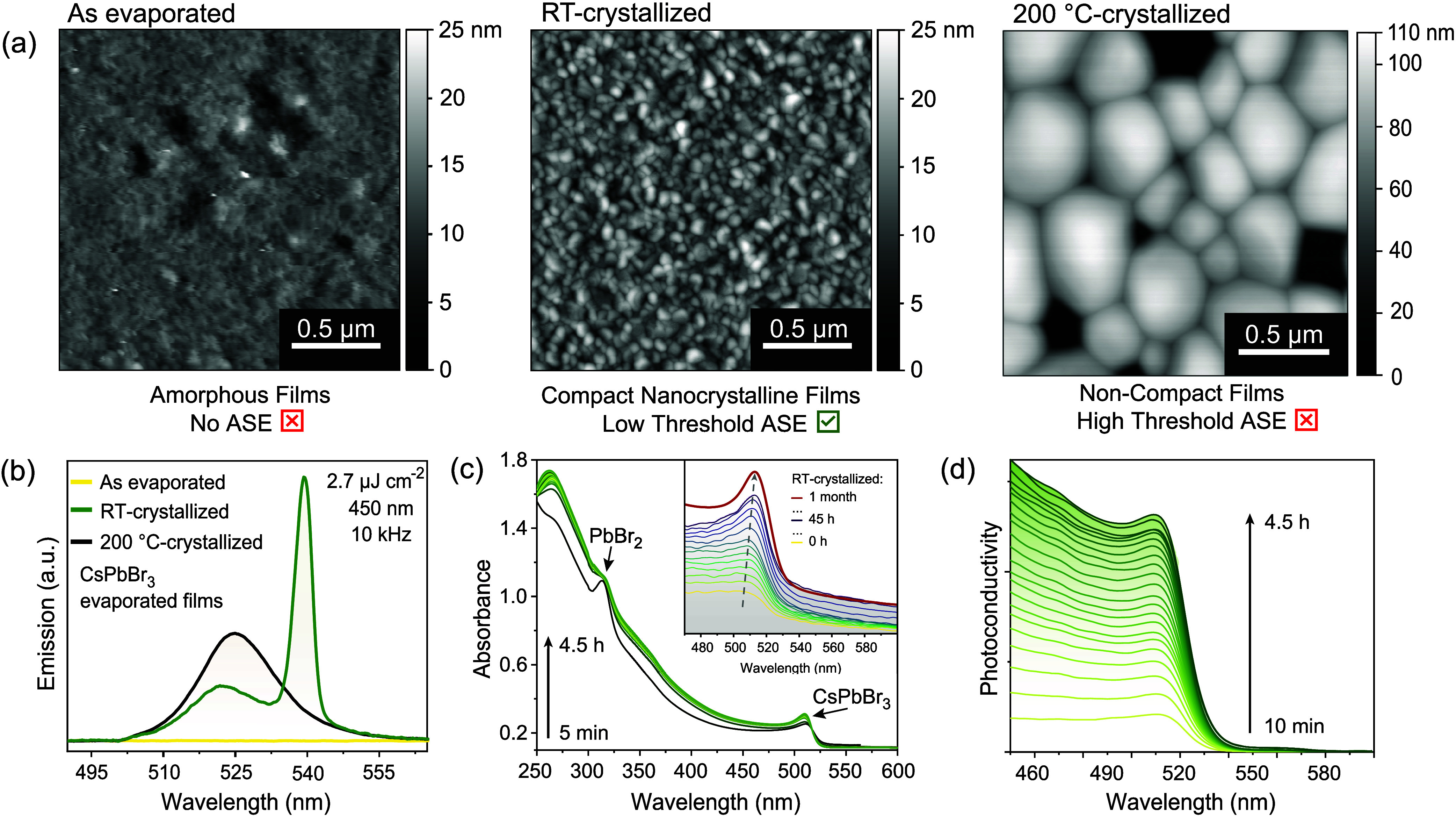
Room-temperature crystallization of single-source thermally evaporated CsPbBr_3_ thin films. (a) AFM images of CsPbBr_3_ evaporated films as prepared (left); after one month of RT-crystallization (middle); and crystallization at 200 °C for >10 min (right). (b) Emission spectra of the corresponding samples from (a) with fs-laser excitation at 450 nm, 10 kHz repetition rate, and 2.7 μJ cm^–2^ energy density. *In situ* recorded: (c) absorption and (d) photoconductivity spectra of CsPbBr_3_ evaporated films during slow crystallization at RT.

The freshly evaporated (amorphous) film is nonemissive in the visible range, whereas both crystallized films display green photoluminescence (PL) at RT, with a peak at around 523 nm, coarsely comparable to the prototypical green PL of weakly confined CNCs (Figure 1b). The RT-crystallized film exhibits a broad PL band of lower intensity, accompanied by an additional narrow and intense red-shifted band at 540 nm. The latter feature appears above a certain threshold (*ca*. 2 μJ cm^–2^) in excitation density and is related to the ASE process.^13^ The films crystallized at 200 °C also exhibit ASE, albeit at a substantially higher threshold (*ca*. 30 μJ cm^–2^) than those at RT. This can be explained by the specific film morphology where the high-T-crystallized exhibit voids and other morphological inhomogeneities, which likely induce scattering and, consequently, optical losses.

The slow crystallization process of CsPbBr_3_ films under ambient conditions, was monitored using absorbance and photoconductivity spectroscopy (Figure 1c,d, S2), as well as AFM (Figure S3 and Video S1), immediately after the deposition to track the changes resulting from the film’s transformation under ambient conditions. AFM images clearly show the formation of nanocrystalline grains consuming amorphous materials and partially some neighboring grains. Such an effective lateral material transfer results in a desirable compact and flat film of high optical quality, with an RMS roughness of only 3.3 nm, as determined by AFM (18 nm when crystallized at 200 °C)

The absorption band at 314 nm has previously been associated with PbBr_2_ and is seen to decrease simultaneously with the growth of CsPbBr_3_ grains within at least 4.5 h (Figure 1c).^59^ Hence, thermal evaporation of the single-crystal CsPbBr_3_ precursor does not immediately lead to CsPbBr_3_ thin films of perfect stoichiometry. The initial film appears to comprise clusters of CsPbBr_3_ and considerable fractions of PbBr_2_ and CsBr, which only slowly crystallize into the final CsPbBr_3_ perovskite grains. This growth is highlighted by the formation and subsequent red shift of an excitonic feature over time (Figure 1c, inset, and Figure S2) from its initial position near 504 nm, corresponding to a size of *ca*. 8 nm for CsPbBr_3_,^3,60^ to 512 nm, which relates to a size of *ca*. 25 nm. The position of the excitonic feature after 45 h (∼2 days) corresponds to a weak confinement regime and is associated with the formation of nanocrystalline CsPbBr_3_ grains. After the initial growth period the absorption spectra show an increase in the excitonic absorption feature near 512 nm. This conclusion is confirmed by *in situ* photoconductivity spectra (Figure 1d), equally revealing an increase in bulk and excitonic features of CsPbBr_3_.

### Structural Characterizations

Thermally evaporated and RT-crystallized CsPbX_3_ films are optically transparent (Figure 2a) with a homogeneous flat surface and tightly packed nanocrystalline grains with an average size of 100–200 nm, as seen by SEM (Figure 2b). The cross-sectional SEM images (inset to Figures 2c and S4) reveal columnar crystals perpendicular to the substrate that span the entire width of the film. To determine the resulting phase composition of the nanocrystalline grains, we performed X-ray diffraction (XRD) measurements on the films, which revealed only a minimal set of strong reflections corresponding to the [001] direction. The strongest reflections were assigned to the (002) and (004) planes using Rietveld refinement. The lack of reflections of other crystallographic directions, compared to the starting material, can be explained by the preferential orientation of the domains on the substrate. Most of the grains grow along the c-direction of the orthorhombic (*Pnma*) CsPbBr_3_ (Figure 2c), orthogonal to the substrate surface which may result from the slow crystallization at RT.^56,61,62^ Temperature has previously been shown to influence the shape of CsPbBr_3_ crystals, where the growth at lower temperatures promoted an elongated shape.^63,64^ The broadening of the individual reflections indicates a reduction of the grain sizes (to about 50–60 nm) between the powdered crystal and the evaporated film (Figure S5). To exclude the presence of other crystalline phases, 800 nm-thick CsPb­(Br/Cl)_3_ films were prepared and scratched from the substrates for analysis with powder-XRD (Figure S6). The results confirm that no other phases or degradation products are present in the films.

**Figure 2:**
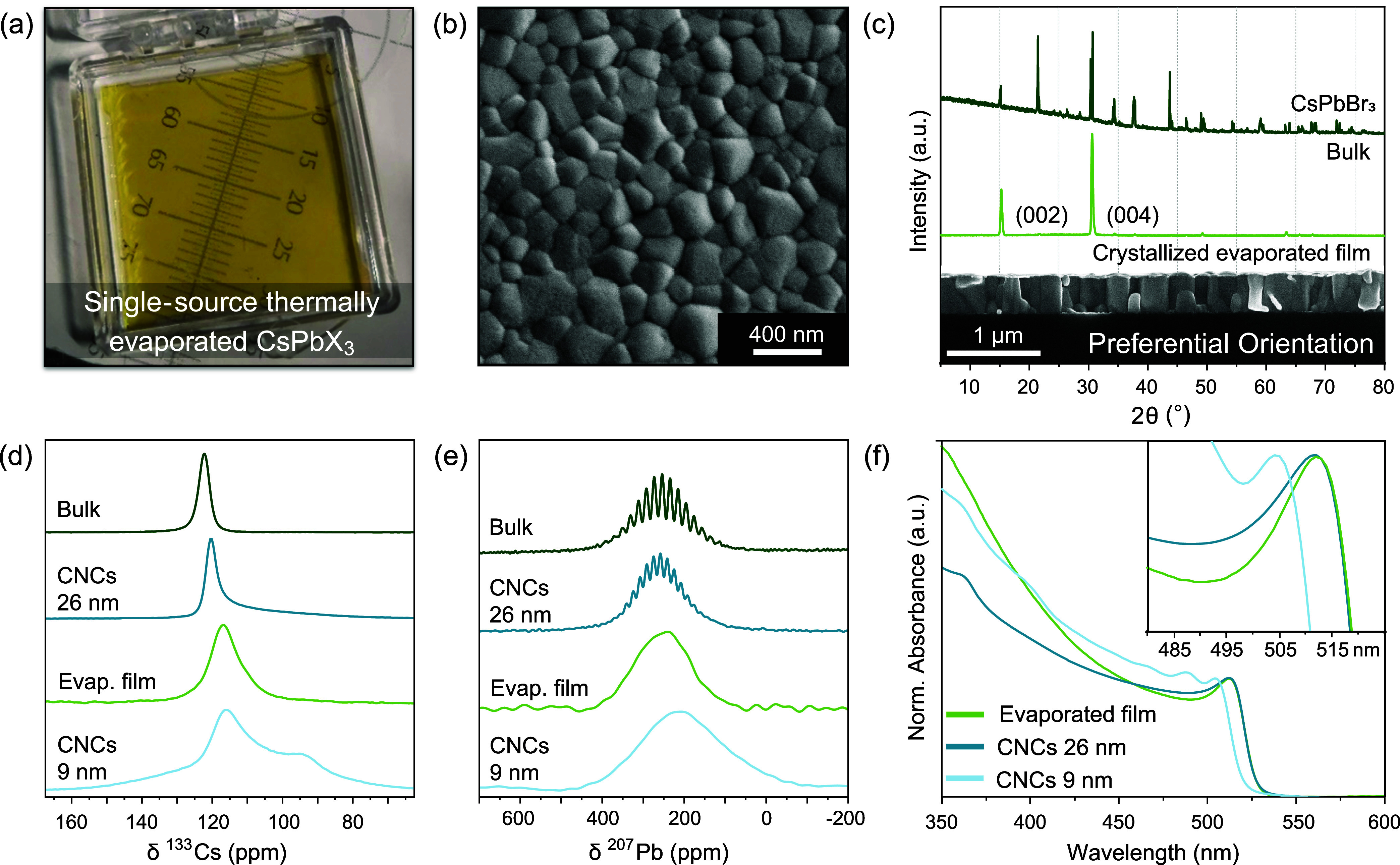
Structural characterization and evidence of large local structural disorder in evaporated nanocrystalline grains. (a) Optical image of a 100 nm-thick thermally evaporated CsPbBr_3_ thin film on a soda-lime glass substrate. (b) SEM top image of a 400 nm-thick evaporated CsPbBr_3_ film. (c) XRD patterns of the bulk (crystalline powder; dark-green line) and a 800 nm-thick RT-crystallized evaporated CsPbBr_3_ film (light-green line). The inset shows a cross-section SEM image of the 400 nm-thick CsPbBr_3_ film. (d) ^133^Cs MAS NMR spectra of bulk (dark-green line), CNCs (size of 26 and 9 nm; dark- and light-blue lines, respectively), and thermally evaporated CsPbBr_3_ films (light-green line) obtained at 16.4 T. (e) ^207^Pb MAS NMR spectra of the corresponding samples from (d). (f) Optical absorption spectra of CNCs (size of 26 and 9 nm) drop-cast and thermally evaporated CsPbBr_3_ film, the absorbance spectra were normalized to the values at excitonic features. Inset: absorbance spectra in the bandgap region.

To conduct an in-depth exploration into the local structure of the obtained nanocrystalline grains, the ^133^Cs solid-state nuclear magnetic resonance (ssNMR) spectrum (under magic-angle spinning) of the powder obtained from the crystallized evaporated films was compared to the spectra of the bulk material as well as CNCs with mean sizes of 9 and 26 nm. While the starting material exhibits only a single narrow (290 Hz; Full Width at Half Maximum, FWHM) NMR line at 122.5 ppm, the lines of the CNCs and thin film are more complex and shifted (Figure 2d). Both spectra of the CNCs exhibit additional features, including a second peak at lower chemical shifts and a shoulder at higher chemical shifts, which are particularly pronounced for the smaller CNCs. Additionally, the spectra of both the CNCs and crystallized evaporated films show increased broadening. These features have been previously assigned to surface distortions induced by surface-bound ligands, while increasingly broader lines were associated with the decreased size of the CNCs.^65^ Such ligand-related signals are not observed for the evaporated film. The most significant observation is the gradual decrease in chemical shift, from the bulk to the 26 nm CNCs, then to the evaporated film, and finally to the smaller 9 nm CNCs. The observation was previously linked to the lattice expansion experienced by smaller crystalline grains, resulting in a change in the immediate chemical environment around Cs.^65^ Based on these observations, we suggest that evaporated nanocrystalline films exhibit a local structure significantly different from bulk CsPbBr_3_ and closer to CNCs. Similarly, the absence of a visible multiplet (*i.e*., ^1^J_Pb–Br_)^66^ due to the broadening of the ^207^Pb ssNMR line for the evaporated film (Figure 2e) provides an additional indication of the increased degree of disorder naturally found for nanocrystalline morphologies. While the grain sizes of the evaporated films observed in AFM and SEM images appear too large to experience quantum confinement, the structural disorder unveils their close relation to CNCs. Both the ^133^Cs and ^207^Pb NMR are consistent with previously reported spectra of CsPbBr_3_ and do not show the presence of residual precursors or degradation products.^67–69^

The absorption spectra of the CsPbBr_3_ CNC and thermally evaporated films show pronounced excitonic features (Figure 2f). These have been previously linked to the effects of quantum confinement.^13^ The absorption peak of the evaporated film is the same as that of the 26 nm drop-cast CNC film. Such optical absorption properties are consistent with the similar local structures of all samples, as inferred from NMR. We conclude that local structural disorder-confined excitons in a nanoscale volume are weakly confined. As shown below, this is a crucial aspect for rationalizing their high optical gain and low-threshold ASE.

### Amplified Spontaneous Emission from 100 nm-Thick CsPbBr_3_ Evaporated Films

The ASE phenomenon was investigated using a 250 fs-laser excitation at 450 nm. At excitation densities below 2.2 μJ cm^–2^ (ASE threshold), only a spontaneous emission (*i.e*., PL) band with a maximum at 522 nm was observed (Figure 3a). An additional narrower, red-shifted band appears at 540 nm when the excitation density increases beyond the ASE threshold (Figure 3b). The ASE threshold indicates the excitation density at which the population of ground and excited states is inverted.^13,70^ Notably, the minimal ASE threshold in our experiments was observed for CsPbBr_3_ thin films prepared *via* single-source evaporation. In contrast, the mean ASE thresholds of coevaporated films (from two sources of CsBr and PbBr_2_) were ∼2.5 times larger compared to the single-source thermally evaporated films (Figure S7).

**Figure 3:**
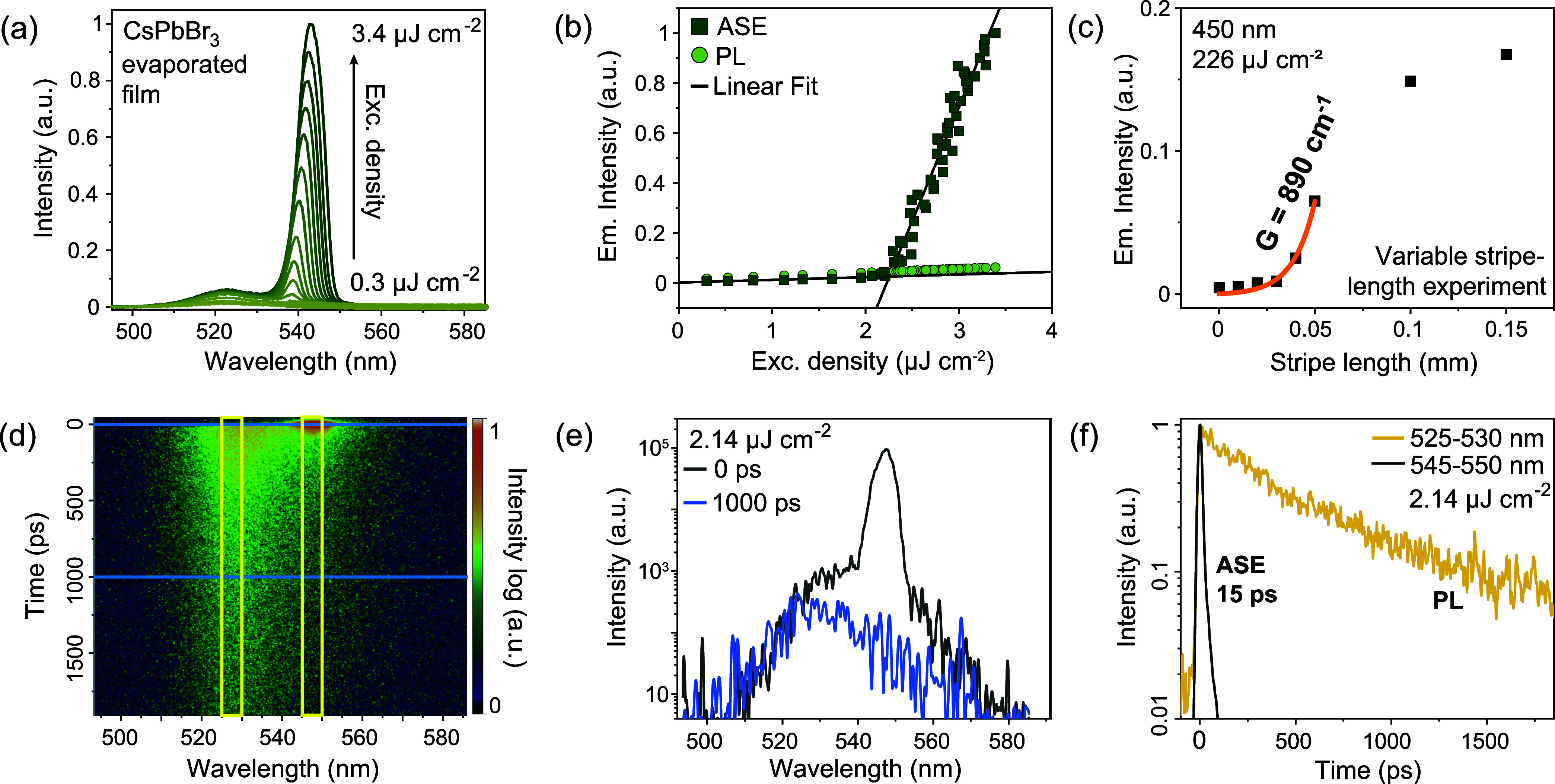
ASE from the 100 nm-thick RT-crystallized evaporated CsPbBr_3_ film under ambient conditions. (a) Emission spectra of evaporated CsPbBr_3_ film at different excitation densities. (b) Emission intensity *vs.* excitation density at the emission wavelengths of the ASE (about 540 nm; dark-green markers) and PL (525 nm; light-green markers) peaks, respectively. The ASE threshold was determined as 2.2 μJ cm^–2^ by the intercept of the linear fits (black line) below and above the threshold. (c) Variable stripe-length experiment for the CsPbBr_3_ evaporated film (black markers) and the corresponding fit for the net modal gain parameter determination (orange line). (d–f) Time- and wavelength-resolved streak camera images (d) under 450 nm laser excitation (2.14 μJ cm^–2^, 10 kHz, 250 fs), with (e) corresponding PL spectra at 0 ps (dark-gray line) and 1000 ps (blue line), and (f) corresponding normalized time-resolved PL traces in the PL region (spectrally integrated between 525 and 530 nm; yellow line) and ASE region (spectrally integrated between 545 and 550 nm; black line). The streak-camera spectral and temporal cuts displayed in (e, f) are indicated in (d) *via* blue and yellow lines, respectively.

Together with the ASE threshold, the net modal gain coefficient (*G*) is a crucial characteristic of a material with optical amplification performance. It is commonly determined by the variable stripe-length (VSL) method, where a cylindrical lens focuses the laser beam into a narrow stripe shape on the sample surface.^13,71–73^ The dependence of the intensity of the ASE peak *vs.* the length of the excitation stripe for the 100 nm-thick CsPbBr_3_ film is shown in Figures 3c and S8. For stripe lengths up to 0.05 mm, the ASE intensity increases exponentially with increasing stripe length. The threshold region can be fitted using the concept of the net modal gain

1 $$I=\frac{A}{g}\left(e^{\mathrm{GL}}-1\right)$$

where *I* is the ASE intensity, *g* is the optical-gain coefficient, and *L* is the stripe length. Fitting of the experimental results to eq 1 yields a net modal gain of 890 cm^–1^. The typical optical gain of chemically synthesized CsPbX_3_ perovskite single crystals, cuboids, nanowires, and nanoplatelets has been reported to be *ca*. 10^3^ cm^–1^,^74^ with an exceptional record-high value exceeding 10^4^ cm^–1^.^50^ This demonstrates the excellent optical gain performance of our evaporated CsPbX_3_ film, as further shown.

Another experimental proof that efficient ASE took place in our experiments was obtained by performing ultrafast PL spectroscopy with a streak camera (time resolution 1 ps). While the decay of spontaneous emission exceeds 500 ps, the time evolution of the ASE band exhibits an ultrafast decay of *ca*. 15 ps (Figure 3d–f), evidencing the occurrence of the stimulated emission process.

### Spectral Tunability of LHPs Evaporated Films *via* Compositional Engineering

As widely demonstrated in LHP systems, their bandgaps can be adjusted by varying halide composition (Cl^–^ → Br^–^ → I^–^),^1,19,75^ yielding a continuous emission control across the visible spectral range. In solid-state synthesis, growing crystals in a closed ampule preserves the original halide ratios from the starting materials to the final product. The mixed halide (Cl/Br) crystals form a CsPbBr_(3‑*x*)_Cl_*x*_ solid solution. Powder-XRD confirmed the purity of all compounds, showing the same space group (*Pnma*) throughout the full range of Cl/Br variation (Figure 4a). With the addition of chloride, smaller than Br, the reflections shifted to higher angles, indicating a linear contraction of the unit cell.

**Figure 4:**
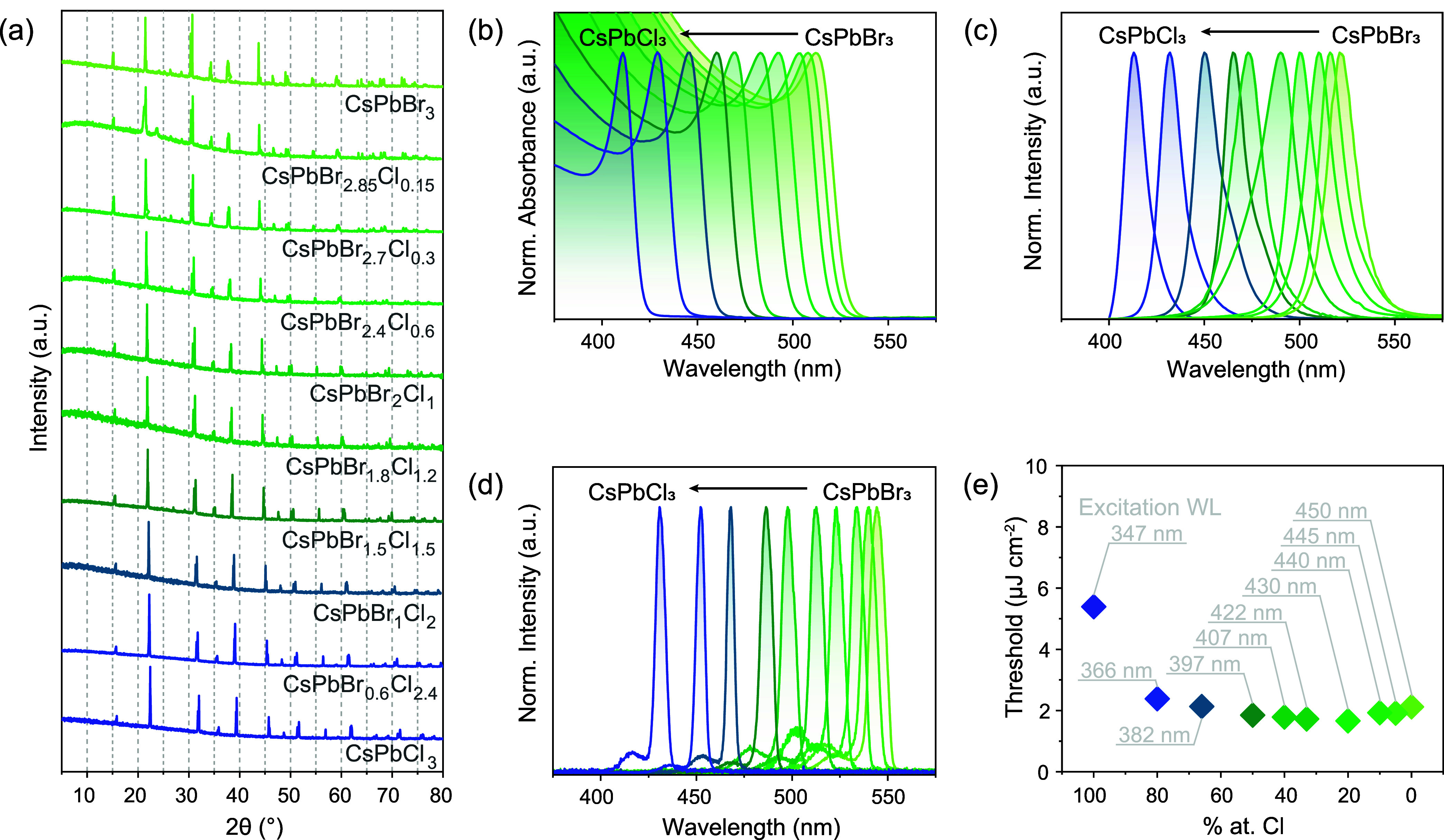
Fine spectral control across the blue-green region *via* compositional engineering of thin films of evaporated CsPbX_3_ (X = Cl, Br). (a) XRD patterns of the CsPbX_3_ (X = Cl, Br) starting materials, showing a linear contraction of the unit cell with increasing amounts of Cl. (b–d) Normalized Absorption (b), PL (c), and ASE (d) spectra of evaporated CsPbX_3_ films. (e) ASE thresholds of CsPbX_3_ films for various halide compositions at the corresponding excitation wavelength (see Table S1).

The excitonic features of CsPbBr_(3‑*x*)_Cl_*x*_ linearly shift with increasing Cl/Br ratio, from 512 to 411 nm, while the ASE band shifts from 545 to 430 nm (Figure 4b–d). The ASE threshold across the mixed-halide series remains almost unaltered at 2 ± 0.4 μJ cm^–2^ for 100 nm-thick CsPbBr_(3‑*x*)_Cl_*x*_ evaporated films, while only the pure chloride sample (*x* = 3) has a higher threshold of 5.4 μJ cm^–2^ (Figure 4e), which is at least 1 order of magnitude lower than the ASE thresholds reported in prior studies on similar materials.^76,77^ To ensure comparable excitation conditions for each stoichiometry, the excitation wavelength was shifted according to the position of the excitonic feature (Table S1 and Figure S9a); meanwhile, the excitation density, pulse width, and repetition rate were kept constant. It may be associated with a decrease in the Stokes shift that is approximately linear with increasing Cl/Br ratio, and thus, optical losses increase in the reabsorption of ASE emission (Figure S9b,c) or a stronger influence of Rayleigh scattering at shorter wavelengths.

### ASE Operational Stability of CsPbBr_3_ Evaporated Compared to CNC Films

For every lasing or optical amplification medium, optical gain stability emerges as an essential and challenging metric that continues to drive research in the field. The stability can be deduced from the temporal decay of the ASE intensity. Here, we compared the operational stability of drop-cast CsPbBr_3_ CNC (ligand-containing) and thermally evaporated (ligand-free) films under pulsed 450 nm fs-laser excitation (6.15 μJ cm^–2^) and ambient conditions (Figures 5a and S10 and S11a). The ASE intensity of the CNC film decreases to 5% of the initial value after 35 million laser shots (*i*.*e*., 1 h of operation). Conversely, the ASE from the evaporated film exceeds 180 million laser shots (*i.e*., 5 h of continuous operation), decreasing to only *ca*. 92% of the initial intensity (Figure 5b,c). Notably, solution-synthesized bulk CsPbBr_3_ films do not exhibit any ASE features even under higher excitation densities (Figure S12a), likely due to the above-mentioned high optical losses inherent to the nonuniform films and enhanced carrier dissociation.

**Figure 5:**
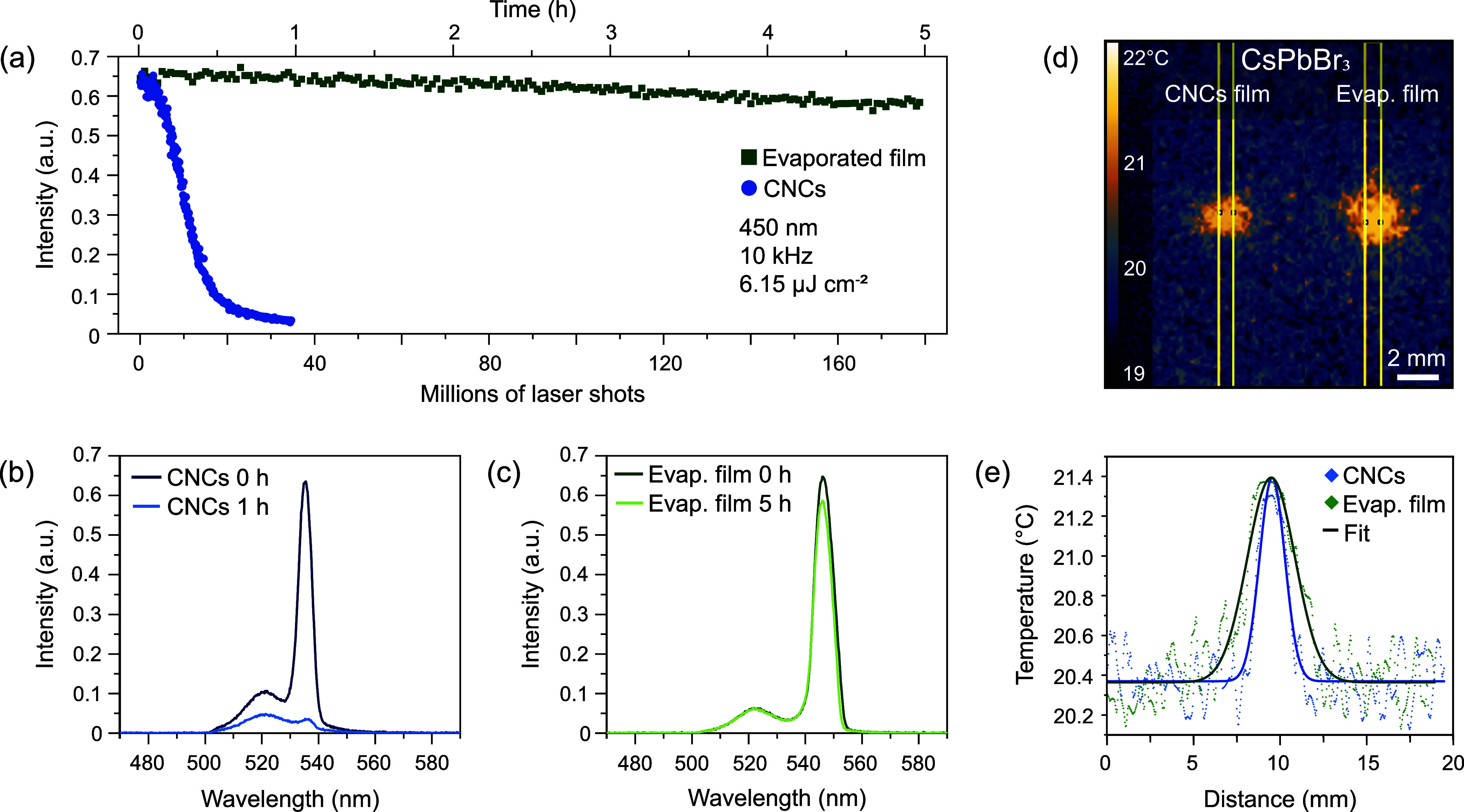
Operational stability of ASE from CsPbBr_3_ thin films (evaporated and drop-cast CNCs). (a) Temporal operational stability of the ASE peak intensity of evaporated (green markers) and drop-cast CNC (blue markers) CsPbBr_3_ films excited at 450 nm (10 kHz; 6.15 μJ cm^–2^). (b, c) Emission spectra of (b) CNC and (c) evaporated thin films at the start and end of the stability test. (d) Thermographic images of a CNC (left) and evaporated (right) CsPbBr_3_ thin films on a thin polymer substrate acquired under the identical conditions to the prior laser excitation. (e) Thermal profiles from the raw data (markers) and Gaussian fits (solid lines) of the drop-cast CNC (blue) and evaporated (green) films extracted from the regions indicated by the yellow lines in (d).

To investigate the origin of the enhanced operational stability of the evaporated films, we prepared both types of samples—evaporated and drop-cast CNC films—on a thin polymer membrane with low thermal conductivity. This allowed us to study heat dissipation in the samples by thermographic imaging. The thermographs under the same excitation parameters revealed a substantially enhanced heat dissipation for the evaporated film inferred from the broader spatial heat profile (3.25 mm FWHM) compared to the drop-cast CNC film (1.72 mm FWHM) (Figure 5d,e). As further support for improved thermal management, we performed operational stability measurements of RT-crystallized evaporated CsPbBr_3_ film under different excitation repetition rates: 1, 10, and 50 kHz (Figure S11b–e). To maintain consistent excitation conditions, all measurements were performed using the 250 fs pulsed laser with a fixed excitation energy of 0.6 μJ and a wavelength of 450 nm. Additionally, to minimize the influence of environmental factors, the experiments were carried out in a nitrogen-filled chamber. The results show consistent ASE performance across the range of repetition rates, indicating that the compact films can effectively manage thermal load without significant degradation in optical gain. This behavior is in line with the expected enhanced dissipation of heat from the pump pulse excess energy is thus consistently repeated with the ligand-free, compact structure of fully inorganic CsPbBr_3_ grains in the evaporated film. Such moderate temperature conditions significantly extend the durability of the operation for the CsPbX_3_ evaporated film observed in our experiments.

## Conclusions

We introduce ligand-free nanocrystalline CsPbX_3_ (X = Cl, Br) thin films obtained *via* single-source thermal evaporation from single-crystal cesium LHPs as a promising optical-gain material with long-term stable performance under ambient conditions. Postdeposition, evaporated films undergo a slow conversion under RT conditions into defined nanocrystalline grains with preferential orientation, forming an overall compact film of high optical quality with an RMS roughness of ∼5 nm. A 100 nm-thick evaporated CsPbBr_3_ film exhibits a low ASE threshold of ∼2.2 μJ cm^–2^ with a high net modal gain of 890 cm^–1^. The ASE spectrum can be continuously tuned *via* the halide composition, spanning the spectral range from 430 to 545 nm as the composition transitions from CsPbCl_3_ to CsPbBr_3_. Importantly, evaporated films exhibit remarkable operating stability under ambient conditions, with a mere 8% drop in ASE intensity after 180 million laser shots (*i.e*., 5 h of continuous operation), clearly outperforming CsPbBr_3_ CNC thin films, which lose 95% of their ASE intensity after just 1 h of operation. While the local structure appears comparable in both materials (with a high degree of structural disorder typical of nanocrystalline compounds, as inferred from NMR), we suggest that the absence of thermally insulating ligands and compact morphology in evaporated thin films greatly facilitates heat dissipation and, hereby, long-term operational stability. Overall, such thermally evaporated compact nanocrystalline films of CsPbX_3_ appear promising, stable, and wavelength-tunable optical gain media for lasing applications at RT or on-chip light amplification.

## Methods Section

### List of Chemicals

Lead­(II) bromide (PbBr_2_; 99.998%, Alfa), trioctylphosphine oxide (TOPO; 90%, Strem), cesium carbonate (Cs_2_CO_3_), diisooctylphosphinic acid (DOPA; 90%, Sigma-Aldrich), oleic acid (OA; 90%, Sigma-Aldrich), *n*-octane (>99%, for synthesis, Roth), *n*-hexane (>97%, HPLC, Sigma-Aldrich), toluene (anhydrous >99.8%, Sigma-Aldrich), mesitylene, acetonitrile, ethyl acetate, Cs-EA (erucic acid, 90–95%, Sigma-Aldrich), dimethylformamide (anhydrous >99.8%, Sigma-Aldrich), dimethyl sulfoxide (anhydrous >99.9%, Sigma-Aldrich).

2-octyl-1-dodecyl phosphoethanolamine (C_8_C_12_ PEA) was used as a capping ligand for CNCs and synthesized as described in previous literature.^67^ All the chemicals were used as received without further purification. Solvents were dried with molecular sieves (2 Å) before use.

### Stock Solutions of Precursors

PbBr_2_-TOPO precursor, 0.067 M. PbBr_2_ (0.2 mmol) and TOPO (1 mmol), dissolved in *n*-octane (2.5 mL) at 120 °C on a hot plate until a clear solution is obtained. Cs-DOPA precursor, 0.02 M. Cs_2_CO_3_ (0.3 mmol), and DOPA (1 mL) were mixed in *n*-octane (2 mL) and heated to 120 °C until the salt dissolved and gas evolution ceased. After cooling to RT, the solution is diluted with *n*-hexane (27 mL) to reach 0.02 M concentration.

### Synthesis of CsPbX_3_ (X = Cl, Br) Single Crystals

Single crystals of CsPbBr_3‑x_Cl_*x*_ (*x* = 0 ÷ 1) were grown using the vertical Bridgman method. High-purity (5N) CsBr, CsCl, PbBr_2_, and PbCl_2_ were mixed in a stoichiometric ratio and sealed in an evacuated quartz ampule. Perovskite polycrystalline materials were synthesized in a muffle furnace by melting raw materials and holding the melt at ∼650 °C for 10 h to ensure complete homogenization. Then, ampules were placed in a two-zone Bridgman furnace. The hot zone was kept at a temperature range of 650–690 °C, while the cold zone was set to a temperature range of 400–450 °C. The growth process was carried out at a 0.5–1.5 mm h^–1^ speed. After the crystallization, the ingots were slowly cooled to RT for a few hours. As a result, homogeneous transparent single crystals were obtained.

### Fabrication of Single-Source Thermally Evaporated CsPbX_3_ (X = Cl, Br) Films

In our typical fabrication procedure, a Bridgman-grown single crystal of CsPbX_3_ (X = Cl, Br) was gently ground into a fine microcrystalline powder in a nitrogen-filled glovebox. The obtained material was placed in a crucible for further thermal evaporation. 25 × 25 mm^2^ 1.1 mm-thick soda-lime glasses were used as substrates, except for the low thermal conductivity polymer film for temperature profile measurement. The substrates were ultrasonically cleaned in Hellmanex III (2% in water), followed by deionized water, acetone, and isopropanol for 15 min at each stage, followed by UV ozone treatment for 10 min. AFM was used to measure the thickness of the fabricated films. Typically, we used a batch of ∼200 mg of starting material to obtain roughly a 100 nm-thick film. The chamber’s vacuum and substrate temperature were maintained at *ca*. 10^–6^ Torr and 20 °C correspondingly during evaporation. The evaporation source temperature was maintained in the range 400–500 °C and regulated by a proportional-integral-derivative controller to maintain a deposition rate of 0.6 Å s^–1^. The substrate rotation velocity was set to 10 rpm. After evaporation, the films were crystallized at RT or annealed on a hot plate in a nitrogen-filled glovebox.

### Synthesis of CNCs

Cs-OA (oleic acid, 90%, Sigma-Aldrich) stock solution was prepared by dissolving 100 mg of Cs_2_CO_3_ into 1 mL of OA and 2 mL of mesitylene at 120 °C in a 40 mL vial, followed by dilution with 27 mL of mesitylene. Cs-EA stock solution was prepared by dissolving 100 mg of Cs_2_CO_3_ into 1.25 mL of EA and 2 mL of mesitylene at 100 °C in a 40 mL vial, followed by dilution with 26.75 mL of mesitylene. PbBr_2_ stock solution was prepared by dissolving 367 mg of PbBr_2_ and 2.15 g of TOPO into 5 mL of mesitylene at 120 °C in a 40 mL vial, followed by dilution with 20 mL of mesitylene. The CNCs were synthesized by modifying the PbBr_2_-TOPO procedure reported in Akkerman *et al*.^3^ by a slow simultaneous injection of Cs-OA or Cs-EA and PbBr_2_-TOPO stock solutions into mesitylene under stirring at 60 °C. After the injection, lecithin or 5k–PS-PEA in toluene was added to the crude solution, followed by washing with acetone, and ethanol or hexane as antisolvents.

Specifically, the CsPbBr_3_ CNCs of 9 nm size were synthesized by the following procedure: PbBr_2_-TOPO precursor (0.5 mL, 0.067 M) was diluted with *n*-hexane (1 mL) and stirred in an open flat-bottom flask on a stirring plate. To this solution, Cs-DOPA precursor (0.25 mL, 0.02 M) is swiftly injected. After 60 s, C_8_C_12_–PEA ligand (10 mg in 0.1 mL of mesitylene) is added. CNCs were purified by the addition of 2 eq. of antisolvent (ethyl acetate:acetonitrile, 2:1 v:v), centrifugation (at maximum centrifugation speed for 30 s), and redispersion of the precipitate in *n*-hexane. The purification was repeated a total of two times.

### Fabrication of Solution-Synthesized Polycrystalline Bulk CsPbBr_3_ Films

CsPbBr_3_ thin films were obtained by spin-coating a 1 M solution of CsPbBr_3_ in 10:1 v:v dimethylformamide: dimethyl sulfoxide onto previously cleaned (see above) soda-lime substrates. The samples were prewetted using dimethylformamide and then dynamically coated with 0.08 mL of the CsPbBr_3_ solution at a spinning frequency of 4000 rpm for 8 s. The sample was subsequently treated with 0.2 mL of toluene as an antisolvent. After coating, the samples were annealed on a hot plate at 100 °C for 10 min under an inert atmosphere.

### Solid-State Magic-Angle Spinning (MAS) NMR Experiments

^207^Pb and ^133^Cs NMR experiments were conducted on a 16.4 T (^1^H at 700 MHz) standard-bore magnet equipped with a 3.2 mm double-resonance MAS probe and an NMR spectrometer (Bruker Avance III HD). Samples were prepared by collecting ∼50 mg of material either by gently removing it from the surface of the samples, precipitating CNCs from the solution, and drying or gently crushing a larger crystal before packing them into a 3.2 mm thick-bottom zirconia rotor. NMR spectra were acquired under magic-angle spinning at 8 kHz using either one-pulse excitation for sensitive bulk samples or an echo sequence with an echo delay of one rotation and an RF field strength of 55 kHz (100 W). Recycle delay times were set to 1.5 times T_1_ as determined by saturation recovery. Typical relaxation times were measured between 0.3 and 1 s for ^207^Pb and 5–10 s for ^133^Cs.

### XRD Measurements

XRD patterns were recorded by a diffractometer (STADI P, STOE & Cie GmbH) equipped with a curved Ge (111) monochromator (Cu Kα_1_ = 1.54056 Å) and a silicon strip detector (MYTHEN 1K, DECTRIS). The patterns were collected in transmission mode (Debye–Scherrer geometry) or reflection mode for powders or films, respectively. The powders were placed in a glass capillary for the powder XRD.

### Scanning Electron Microscopy

Images were obtained using a scanning electron microscope (FEI Magellan 400 FEG-SEM, Thermo Fisher Scientific) at an acceleration voltage of 1–2 kV in secondary electron detection mode. The cross-sectional images were recorded using a different microscope (Helios 5 Hydra, Thermo Fisher Scientific) at a sample tilt of 0.6° using an acceleration voltage of 7 kV with a current of 100 pA. The samples were prepared by sputter coating a 2 nm Pt/Pd film (CCU 010, Safematic) on the surface, fracturing the glass substrate, and coating an additional 1 nm W film on the freshly prepared cross-section to enhance the electronic conductivity.

### Atomic Force Microscopy

AFM images were obtained by an AFM microscope (NX10 EFM, Park) in noncontact mode (NCM) phase and *z*-height mode with SmartScan software. The scan rate was 0.3 Hz. The scan resolutions were 256 × 256 for 10 × 10 μm^2^ area and 526 × 526 for 2 × 2 μm^2^ area. Sample thicknesses were determined by measuring the step height at regions in where the film had been removed mechanically *via* scratching down to the substrate.

### Absorption Spectra

Ultraviolet–visible (UV–vis) absorbance spectra were recorded using a spectrophotometer (V670, Jasco) equipped with an integrating sphere (ILN-725, Jasco), a deuterium lamp for the UV region (employed at wavelengths below 330 nm) and a halogen lamp for the UV–vis-NIR region (employed at wavelengths above 330 nm). *In situ* absorption spectra were recorded using a second spectrophotometer (UV-2600, Shimadzu), which was also equipped with a deuterium lamp for the UV region (operating at wavelengths below 330 nm) and a halogen lamp for the UV–vis-NIR region (operating at wavelengths above 330 nm). The range near the excitonic feature at the bandgap (inset to Figures 1c and S2) was recorded by a broadband spectrometer (CCS200/M, Thorlabs) with a white LED (D50, LCFOCUS) as a light source.

### Photoluminescence Spectra

The PL spectra under ambient conditions were measured using a spectrometer (FluoTime 300, PicoQuant GmbH) after excitation by a 355 nm ps-laser. Rayleigh-scattered excitation light was discarded by a 400 nm long-pass filter in the emission path. All spectra were corrected for the detector’s spectral sensitivity.

### Amplified Spontaneous Emission

The samples were excited using a tunable femtosecond laser system consisting of a pulsed laser (ORIGAMI XP, 250 fs pulse width, 10 kHz repetition rate) and an optical parametric amplifier (ORIGAMI IRO, NKT Photonics). All experiments were conducted under ambient conditions. The excitation wavelengths (347–450 nm) were chosen according to the position of the excitonic feature in the optical absorption of a particular sample (Table S1). The laser pulse energy was measured using a laser power/energy meter (Centauri, MKS Ophir) equipped with a μJ-measuring head (PE9-ES-C, MKS Ophir). A variable metallic neutral-density filter was used to attenuate the intensity of the laser beam. The beam was focused in a tight stripe shape (∼0.9 × 4.4 mm) using a cylindrical lens with a focal length of 75 mm. The optical emission of the sample was collected from a relatively large solid angle inclined at about 45° to the sample surface, focused into a multimode optical fiber. It was then recorded *via* a broadband spectrometer (CCS200/M, Thorlabs). A long-pass filter in the emission path blocked Rayleigh-scattered excitation light.

### Photoconductivity Spectra

The photoconductivity measurements were performed with illumination from a tungsten lamp dispersed by a monochromator (Acton SP2150, Roper Scientific). A chopper modulated the light at a frequency of 17 Hz. For photoconductivity experiments, a 100 nm-thick CsPbBr_3_ film was evaporated over interdigitated electrodes with 10 μm gaps and a total length of 40 mm. The sample was biased at 1 V using a source meter (236 SMU, Keithley), and the bias was limited to obtain stable dark current in the range of 10 nA. The photocurrent’s amplitude and phase were measured with a lock-in amplifier (SR830, Stanford Research) *via* the observed voltage drop at a load resistance of 10 MOhm. The intensity of the monochromatized light was controlled by a calibrated power detector (UM9B-BL, Gentec-EO).

### Time-Resolved Photoluminescence Decay and Lifetime Measurement

The measurements were performed under ambient conditions using a streak camera (C10910–05, Hamamatsu Photonics). The excitation source was a tunable femtosecond laser system (450 nm, 250 fs laser pulses, and 10 kHz repetition rate, ORIGAMI XP and ORIGAMI IRO, NKT Photonics). The excitation density at the sample was 2.14 μJ cm^–2^.

### Operational Stability Experiment

The experiment was conducted under stable ambient conditions utilizing the femtosecond laser system as the excitation source and the CCD spectrometer to monitor ASE characteristics. All results were obtained at 450 nm laser excitation with 250 fs pulses and a 10 kHz repetition rate. A cylindrical lens focused the laser beam into a stripe with an excitation density of 6.15 μJ cm^–2^.

### Temperature Profiles

The temperature profiles near the excitation stripe at the thin CsPbBr_3_ films (drop-cast colloidal NCs and evaporated) deposited on a polymer film with low heat conductance were captured by a thermal camera (Seek Thermal Compact PRO with an objective upgraded by an additional ZnSe lens) under ambient conditions and 450 nm laser excitation with 250 fs pulses, 10 kHz repetition rate, and an excitation density of 6.15 μJ cm^–2^.

## Supplementary Material





## Notes

CsPbBr3 crystallization AFM video Phase (MP4)
Schematic sample fabrication process, the evolution of the excitonic peak position with time during RT- crystallization, the AFM images of 100 nm-thick thermally evaporated CsPbBr_3_ film during the crystallization process, cross-section SEM images of 100, 400, and 800 nm-thick thermally evaporated CsPbBr_3_ films, XRD patterns of the CsPbX_3_, the comparison of ASE thresholds of single-source and cosource thermally evaporated films, the table of the ASE tunability of 100 nm-thick CsPbX_3_ (X = Cl, Br) films, the raw emission spectra vs the stripe length in the VSL experiment of 100 nm-thick evaporated CsPbBr_3,_ the raw absorption spectra and Stokes shifts of 100 nm-thick evaporated CsPbX_3_ (X = Cl, Br) films, the photos of the drop-cast CNC and evaporated CsPbBr_3_ films, emission spectra and ASE to PL intensity time evolution under 1 kHz, 10 kHz and 50 kHz repletion rate, emission spectra and photos of solution-processed (spin-coated) CsPbBr_3_ films, as well as the video of the detailed crystallization process *via* continuous AFM scanning (PDF)

## Abbreviations

LHPs: lead halide perovskites
CNCs: colloidal nanocrystals
PLQY: photoluminescence quantum yield
ASE: amplified spontaneous emission
RT: room-temperature
AFM: atomic force microscopy
SEM: scanning electron microscopy
NMR: nuclear magnetic resonance
PL: photoluminescence
XRD: X-ray diffraction
ssNMR: solid-state nuclear magnetic resonance
VSL: variable stripe-length
FWHM: Full Width at Half Maximum
