# Supplementary material for: Stable, Room-Temperature, Low-Threshold Amplified Spontaneous Emission from Thermally Evaporated Cesium Lead Halide Perovskites
Source: ACS Nano. 2025 Jul 16;19(32):29216–27. doi: 10.1021/acsnano.5c03771 (PMC12369004; doi:10.1021/acsnano.5c03771)
Supplement: Supplementary file 2 [file nn5c03771_si_002.pdf]

Supporting Information

Stable, Room-Temperature, Low-threshold Amplified Spontaneous Emission from Thermally Evaporated Cesium Lead Halide Perovskites

Yuliia Kominko[1,2‡], Sebastian Sabisch[1,2‡], Andrii Kanak[1,2], Lidiia Dubenska[1,2], Ihor Cherniukh[1,2], Matthias Klimpel[1,2], Xuqi Liu[1,2], Sergey Tsarev[1,2], Simon C. Boehme[1,2], Gebhard J. Matt[1,2], Gabriele Rainò[1,2], Maksym V. Kovalenko[1,2] and Sergii Yakunin[1,2]**

[1] ETH Zürich, Department of Chemistry and Applied Biosciences, Laboratory of Inorganic Chemistry, Vladimir-Prelog-Weg 1, Zürich CH-8093, Switzerland

[2] Empa – Swiss Federal Laboratories for Materials Science and Technology, Laboratory for Thin Films and Photovoltaics, Überlandstrasse 129, Dübendorf CH-8600, Switzerland

Corresponding Authors (*): mvkovalenko@ethz.ch, yakunins@ethz.ch

‡Y.K. and S.S. contributed equally.

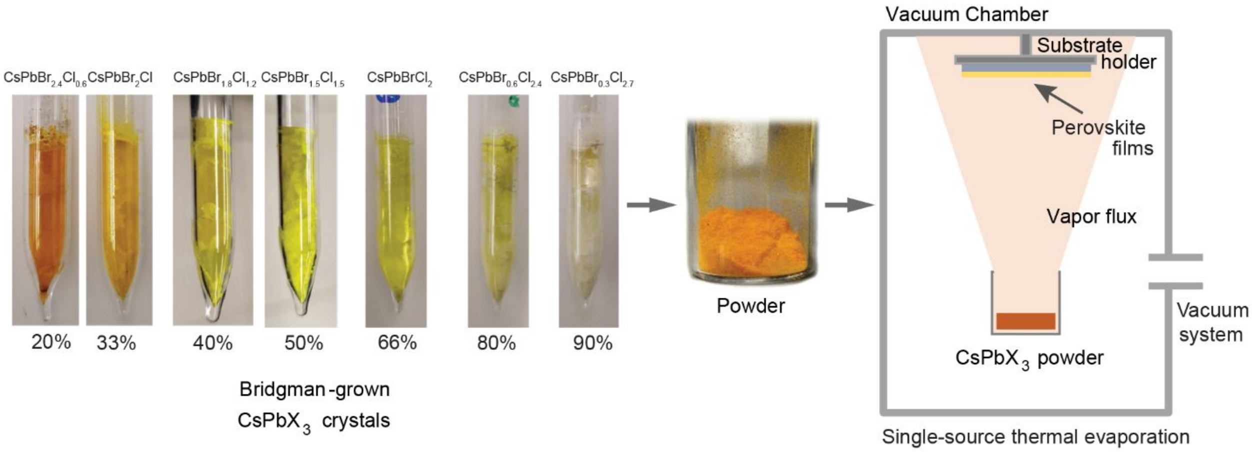

Figure S1. Sample fabrication process by single-source thermal evaporation of $CsPbX_3$ (X=Cl, Br) powders ground from melt-grown crystals.

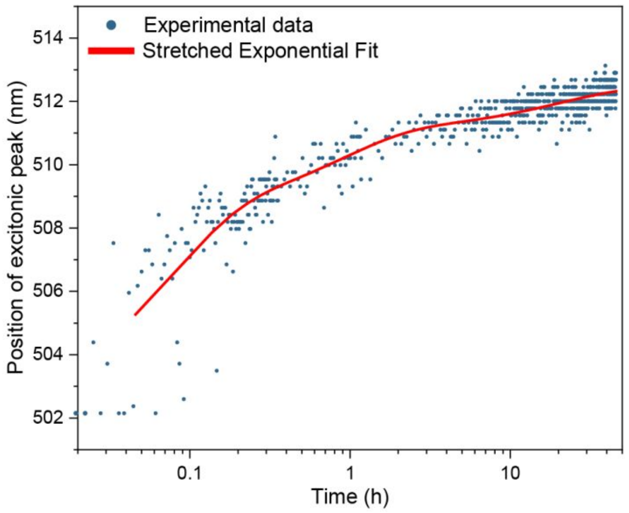

Figure S2. Excitonic peak position *vs* time in absorption spectra for the single-source thermally evaporated $CsPbBr_3$ thin film during the RT crystallization (data from inset to Figure 1c).

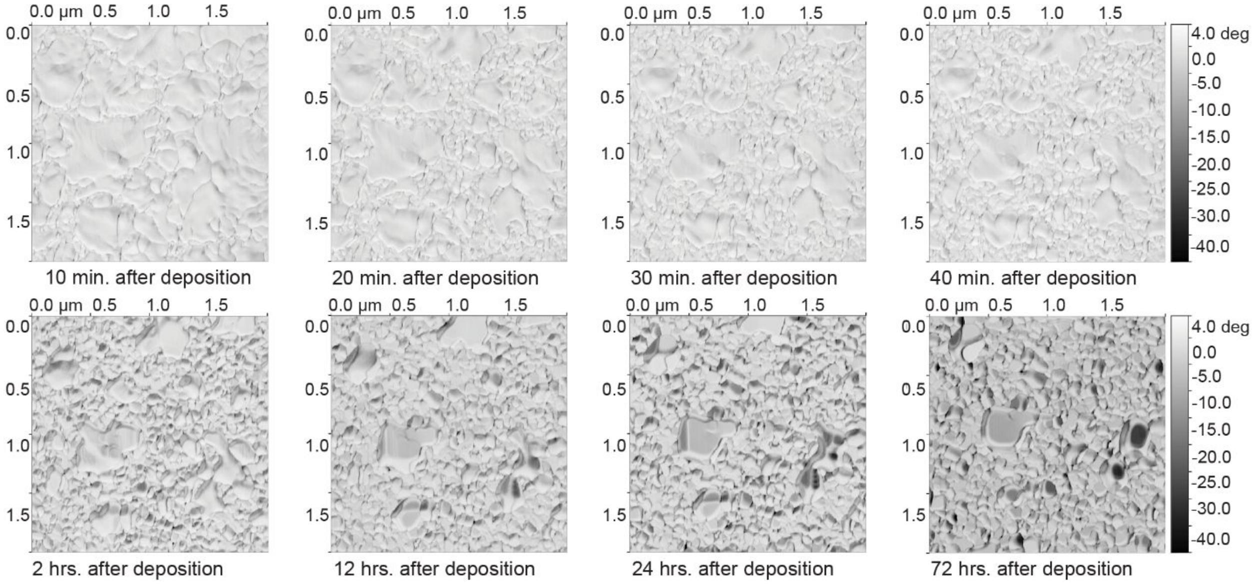

Figure S3. The AFM images of 100-nm thick thermally evaporated $CsPbBr_3$ film during the crystallization process under ambient conditions. The scanning area is 2x2 μm^2. The represented images were recorded in a non-contact mode (forward phase) for the *in situ* video (see Video S1).

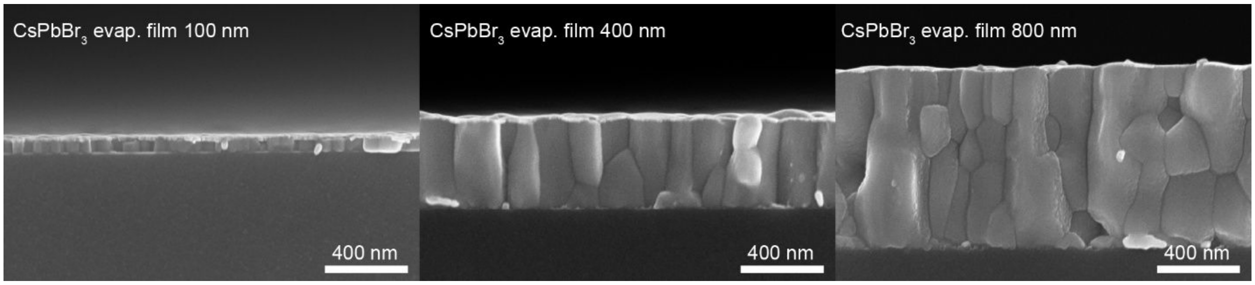

Figure S4. Cross-section SEM images of 100 nm, 400 nm, and 800 nm-thick thermally evaporated $CsPbBr_3$ films show a preferential orientation of the nanocrystalline domains.

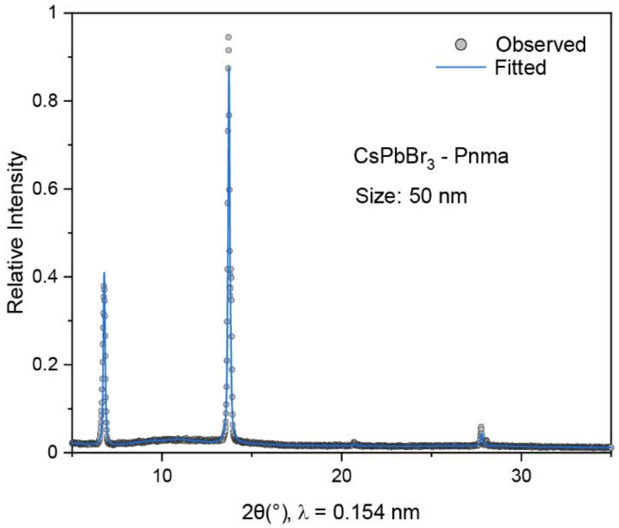

Figure S5. X-ray diffractogram obtained in reflection mode using a molybdenum source. The diffractogram was fitted considering the background of the substrate as well as the preferential orientation and the reduced size of the individual crystallites.

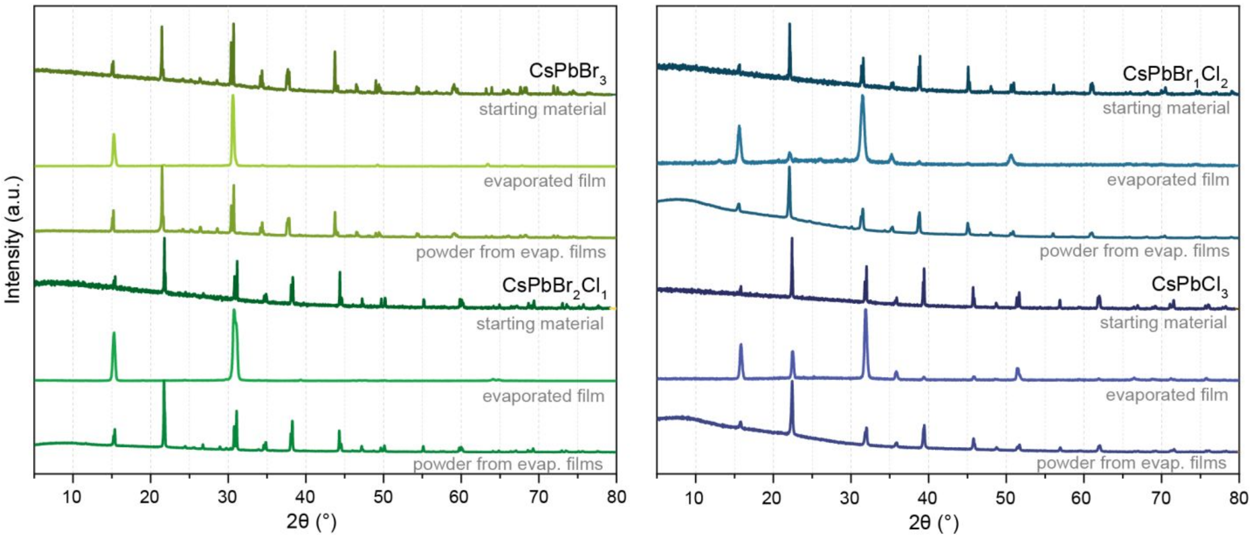

Figure S6. XRD patterns of the $CsPbX_3$ (X = Cl, Br): starting materials, evaporated films, and scratched materials prove a preferential orientation of the perovskite nanocrystalline domains and the preservation of initial stoichiometry in the deposited material.

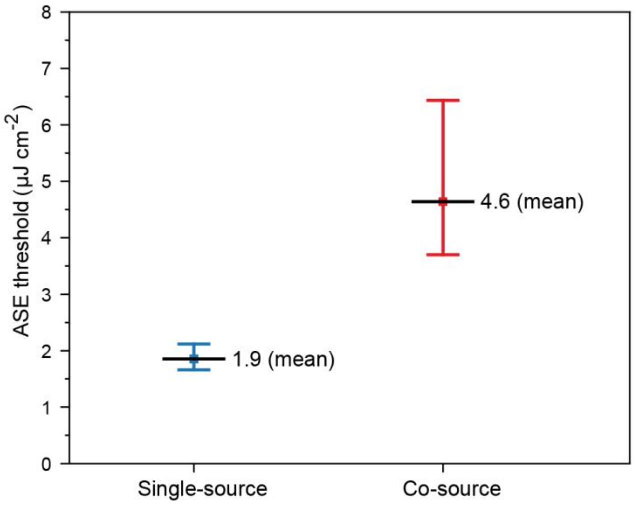

Figure S7. The comparison of ASE thresholds of single-source and co-source thermally evaporated films. The laser excitation wavelength is 450 nm with 250 fs pulses and 10 kHz repetition rate.

Table S1. The ASE tunability of 100-nm thick $CsPbX_3$ (X = Cl, Br) thermally evaporated films under variable excitation wavelength.

% at. Cl	Absorption band WL	Excitation WL	PL peak WL	ASE peak WL
0%	512 nm	450 nm	522 nm	544 nm
5%	508 nm	445 nm	517 nm	540 nm
10%	504 nm	440 nm	513 nm	533 nm
20%	493 nm	430 nm	502 nm	523 nm
33%	483 nm	422 nm	493 nm	512 nm
40%	469 nm	407 nm	478 nm	497 nm
50%	460 nm	397 nm	468 nm	486 nm
66%	446 nm	382 nm	452 nm	468 nm
80%	429 nm	366 nm	436 nm	452 nm
100%	411 nm	347 nm	416 nm	431 nm

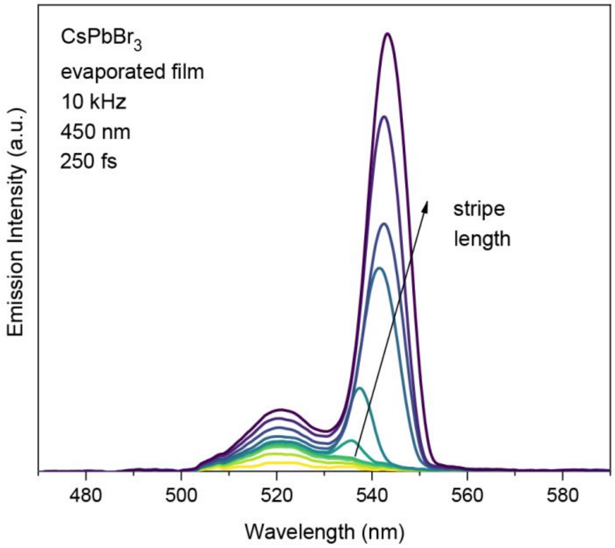

Figure S8. Emission spectra in the VSL experiment with the RT-crystalized $CsPbBr_3$ evaporated film. The spectra correspond to Figure 3c.

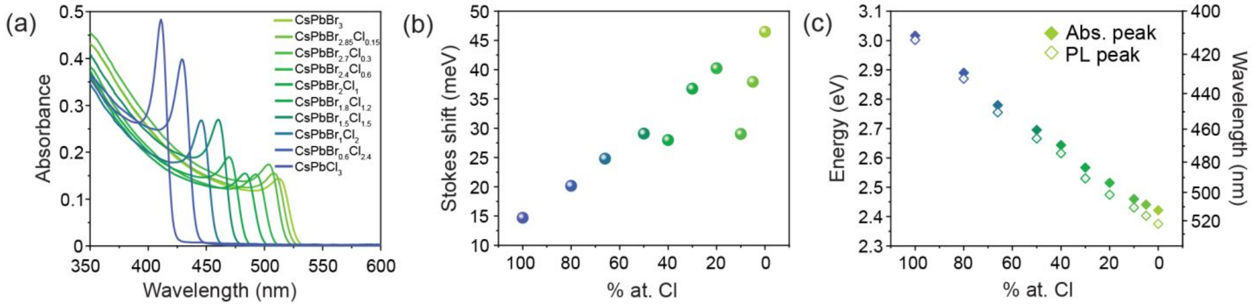

Figure S9. **a**) The raw absorption spectra of *ca.* 100 nm thick evaporated $CsPbX_3$ (X = Cl, Br) films measured with an integrating sphere. **b**) Stokes shift of the corresponding samples. **c**) Absorption and PL peak positions in $CsPbX_3$ films for various halide compositions.

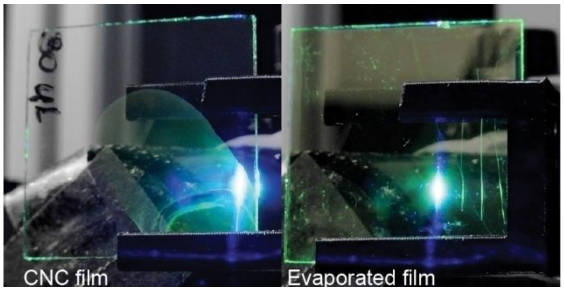

Figure S10. The photos of the drop-cast CNC and evaporated $CsPbBr_3$ films under 450 nm fs-laser excitation.

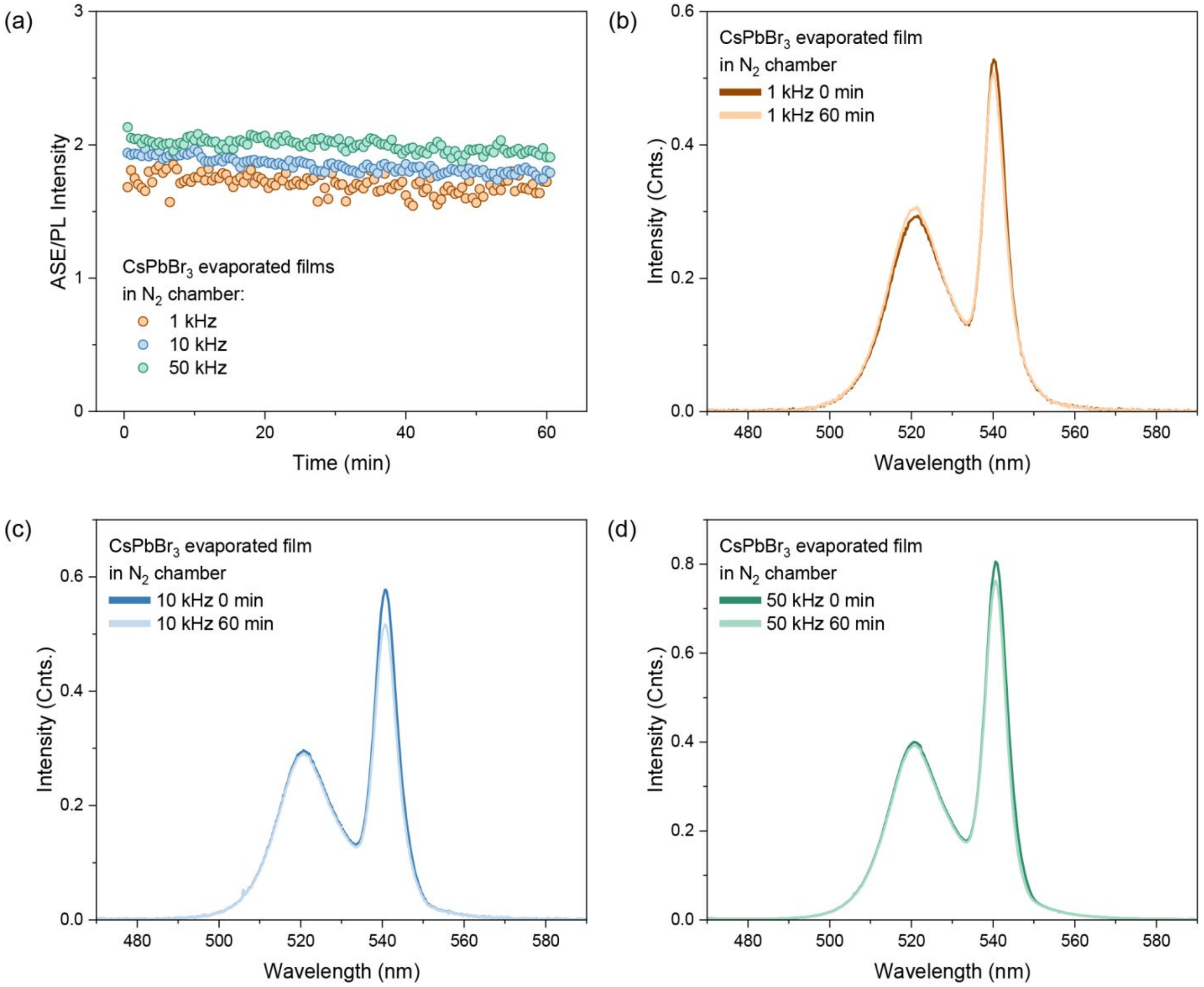

Figure S11. Operational stability of ASE from evaporated $CsPbBr_3$ thin films. **a**) Temporal operational stability of evaporated $CsPbBr_3$ films excited at 450 nm, 250 fs laser excitation (1 kHz, 10 kHz, and 50 kHz, in N_2 chamber). **b-d**) Emission spectra evolution of evaporated $CsPbBr_3$ films in N_2 chamber at 1 kHz (b), 10 kHz (c), and 50 kHz (d).

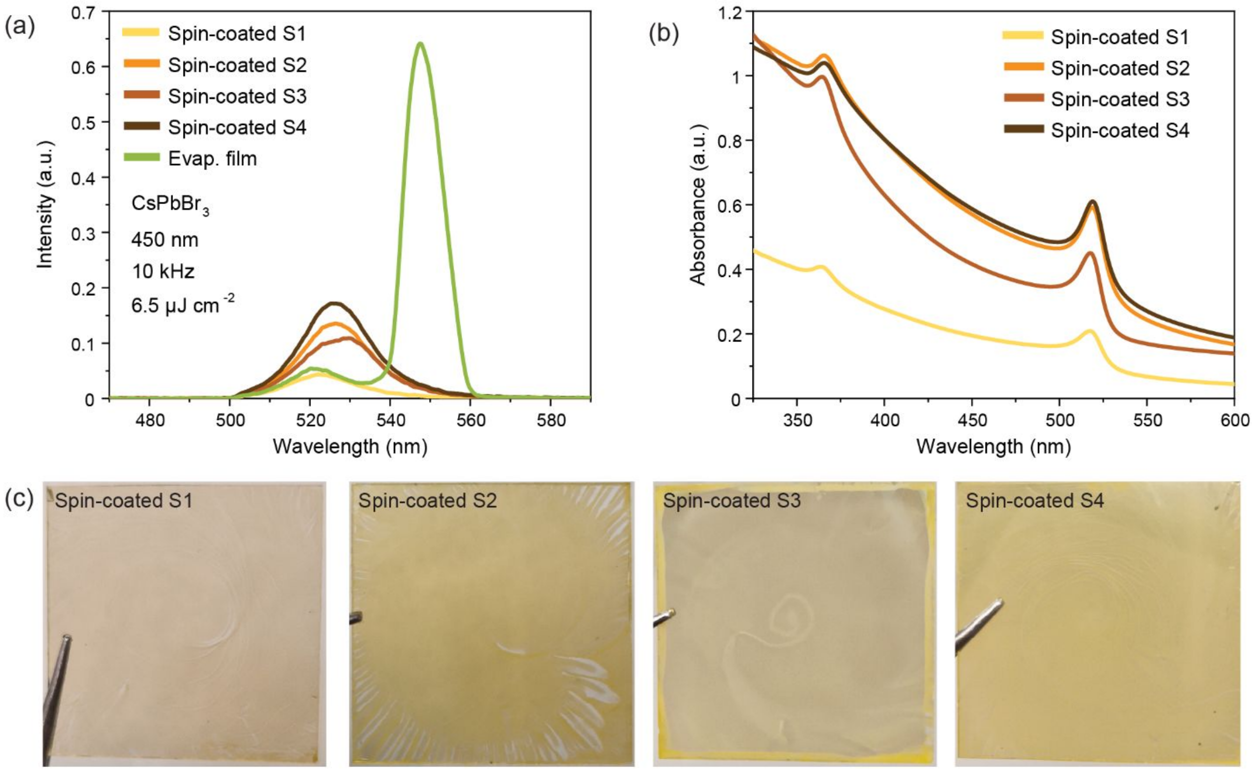

Figure S12. **a**) Emission spectra of evaporated and solution-processed (spin-coated) $CsPbBr_3$ films under 6.15 μJ cm^{-2} fs-laser excitation. No ASE band for solution-processed films was observed. **b**) The raw absorption spectra of solution-processed $CsPbBr_3$ films. **c**) The photos of the corresponding solution-processed samples (fabricated under the same conditions by different operators) demonstrate the irreproducibility of the method.

Video S1. *In situ* AFM images during crystallization. Recorded for 3.5 days (frames are scanned for 10 minutes each), the AFM video represents the crystallization process of a $CsPbBr_3$ film under ambient conditions.
